# Supplementary material for: Skin Wound Healing of the Adult Newt, Cynops pyrrhogaster: A Unique Re-Epithelialization and Scarless Model
Source: Biomedicines. 2021 Dec 13;9(12):1892. doi: 10.3390/biomedicines9121892 (PMC8698868; doi:10.3390/biomedicines9121892)
Supplement: Supplementary file 1 [file biomedicines-09-01892-s001.zip › biomedicines-1504336-supplementary/Supplementary Information & Videos 13 Dec 2021/Supplementary Information_rev.pdf]

Supplementary Information

for

Skin Wound Healing of the Adult Newt, *Cynops pyrrhogaster*: A Unique Re-epithelialization and Scarless Model

Tatsuyuki Ishii, Ikkei Takashimizu, Martin Miguel Casco-Robles, Yuji Taya, Shunsuke Yuzuriha, Fubito Toyama, Fumiaki Maruo, Kazuo Kishi & Chikafumi Chiba

CC: chichiba@biol.tsukuba.ac.jp

KK: kkishi@a7.keio.jp

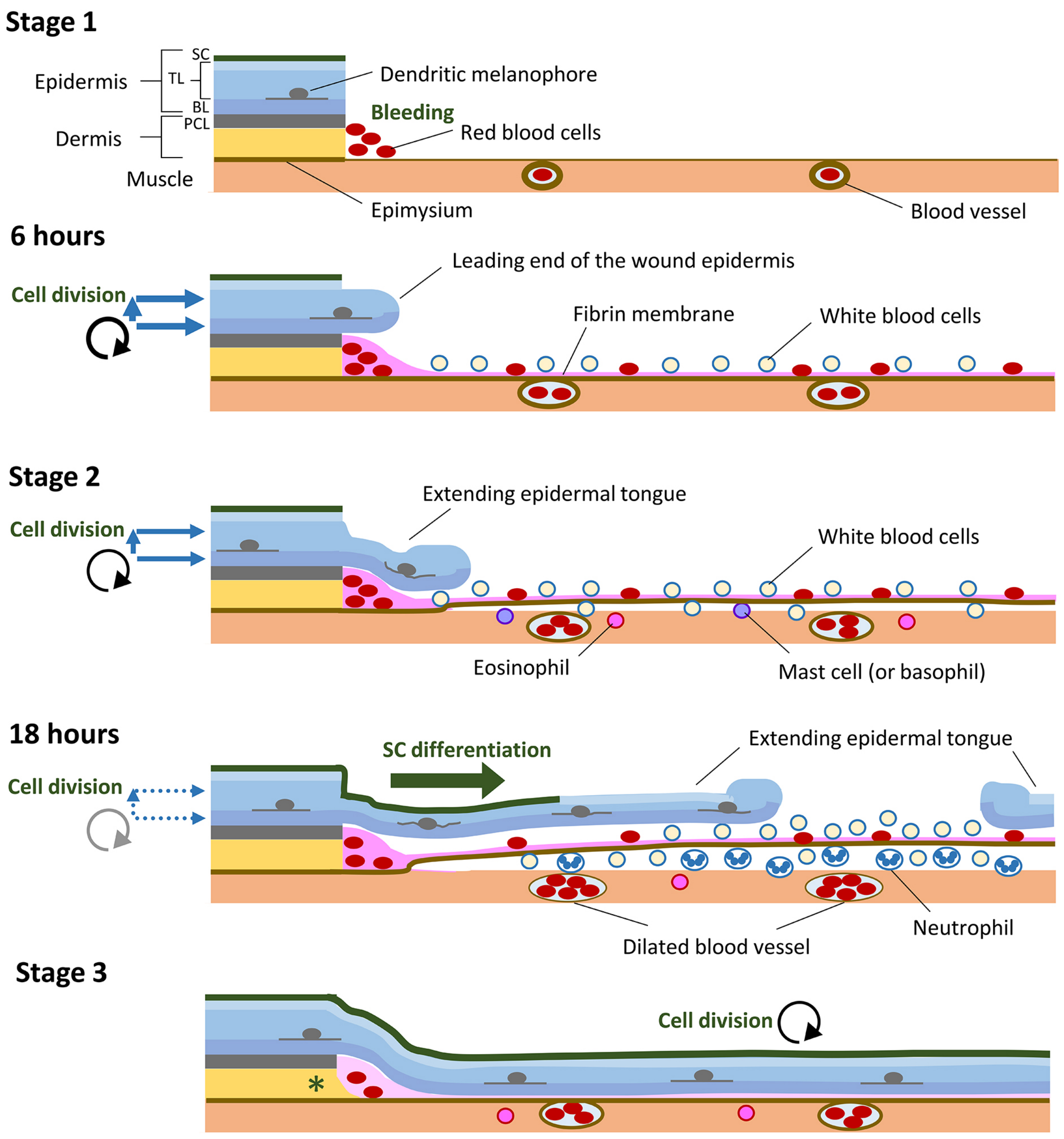

Supplementary Figure S1. The processes of re-epithelialization in the adult newt. Schematics were drawn on the basis of the results in this study (Figures 7–14). After full-thickness skin is excised, the supply of cells to both the basal layer (BL) and transitional layer (TL) is accelerated in the large area of the skin surrounding the wound, and the wound epidermis, which is comprised of cells from the BL and TL, sprouts as early as 6 hours with the leading end (a streamlined shape in the transverse section), while leaving the stratum corneum (SC) and an outer part of the TL behind. The wound epidermis lands on the wound bed within 12 hours (Stage 2) and continues to extend, as the extending epidermal tongue, over the surface of the wound bed as if it glides on the soft substrate of tissue fluid containing white blood cells over the fibrin-like membrane or epimysium tissue. While the extending epidermal tongue further increases its area to cover the wound bed, the SC starts to differentiate from the proximal end of the epidermal tongue. At the stage when coverage on the wound bed reaches about two-thirds of the distance between the wound margins (18 hours), the number of neutrophils obviously increases under the epimysium tissue in and around the uncovered wound bed. Since the wound bed has become completely covered by the wound epidermis by 48–70 hours (Stage 3), cell division in the skin surrounding the wound, as well as inflammatory reactions represented by the gathering of neutrophils, return to normal levels. The basal stem cells in the wound epidermis begin cell division. Dermal reconstruction is about to start from the place of the wound margin

(asterisk). Note that the dendritic melanophores were not present in the dorsal-lateral to ventral skin with a color pattern (Figure 1i, j). PCL: pigment cell layer. For more details, see text.

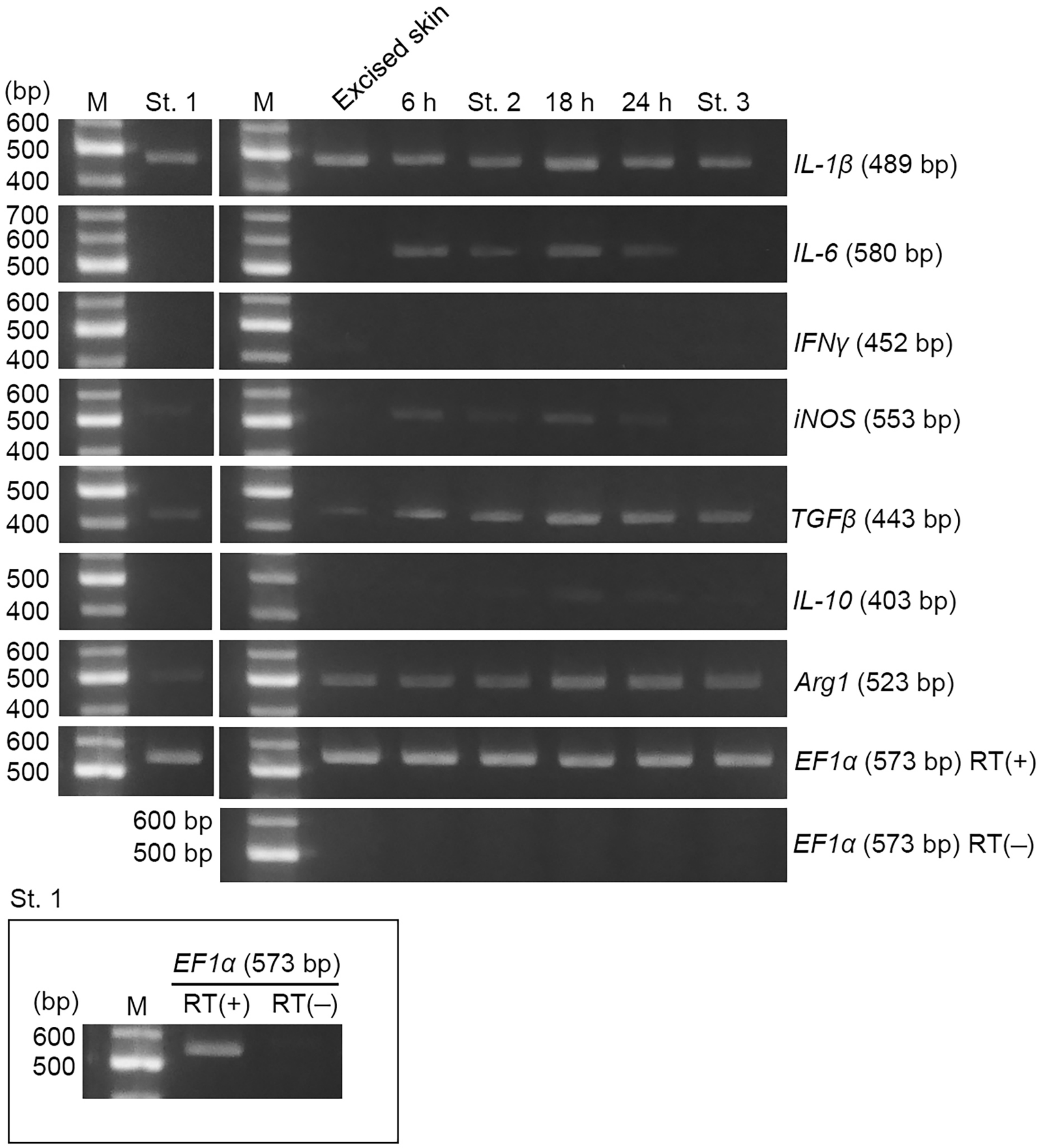

Supplementary Figure S2. PCR results for inflammation-related genes. Sample images of electrophoresis are shown. Immediately after full-thickness skin was excised from the dorsal part of the forearm, the excised skin and subcutaneous tissue up to a few millimeters deep from the wound surface were collected as samples for Excised skin and Stage 1 (St. 1), respectively. After the excision of full-thickness skin, samples of the wound at 6 hours, Stage 2 (12 hours), 18 hours, 24 hours, and Stage 3 (96 hours) were also collected. These samples contained the growing or grown wound epidermis as well as the subcutaneous tissue in the range between the wound surface and a depth of a few millimeters. M: size marker. IL-1β: interleukin-1 beta; IL-6: interleukin 6; iNOS: inducible nitric oxide synthase; IFNγ: interferon gamma; TGFβ: transforming growth factor beta; IL-10: interleukin 10; Arg1: arginase 1; EF1α: elongation factor 1 alpha. The values in parentheses are the lengths of the PCR products. For PCR cycle number, see Supplementary Table S2. RT(+) and RT(–) indicate the presence and absence of reverse transcription in the process of cDNA construction. The data in the box (St. 1) shows a sample set of PCR results for EF1α with cDNA samples of RT(+) and RT (–) at Stage 1.

Supplementary Table S1. Genes examined in this study. *IS*-transcript: contig ID found in the comprehensive transcriptome data base 'TOTAL' in the sequence resource site 'IMORI' for *C. pyrrhogaster* (http://antler.is.utsunomiya-u.ac.jp/imori/) [23]. The sequences of *TGFβ1* and *EF1α* have been reported previously [23].

Gene name	Abbreviation	*IS*-transcript	References
Interleukin-1 beta	IL-1β	comp500726_c0_seq3	45–48
Interleukin-6	IL-6	comp281857_c0_seq1	46–50
Interferon-gamma	IFN-γ	comp467643_c0_seq1	47, 48
Inducible nitric oxide synthase	iNOS	comp495137_c0_seq1	16, 51
Arginase 1	Arg1	comp457482_c1_seq2	47, 51
Interleukin-10	IL-10	comp501686_c0_seq3	46–48, 52

In this study, the expression of IL-1β, IL-6, IFN-γ and iNOS were examined as inflammatory cytokines, and TGFβ [46–48], IL-10 and Arg1 were examined as anti-inflammatory cytokines (references are listed in the right-hand column).

Supplementary Table S2. PCR primer sets and amplicon size.

Gene	Primer sequences		Amplicon size (bp)	Cycle number
	Forward (5′–3′)	Reverse (5′–3′)		
EF1α	GACCTTTGCCCCCAGTAACGTAACCAC	ACTGGGTGTTGCTGGCGCTACTTCTTG	573	25
IL-1β	CCAATGCCTCACGGCAAGTCAAGTT	GCTCCGTTAGTTCTCCTTGCACACACTGA	489	28
IL-6	CATGCCTTCTCCACTGAGAGCATCCAGA	ACGAGCAGTCACTCTCTCTGTCCAGACTT	580	30
IFNγ	GCTCCTCGAGCATCTGTCATCTTCCAG	AGCAAGGCTATCTCCTGGAAGATGCTG	452	32
iNOS	CAATCAGCAAGCACGGTTGCGATGGAT	GATCAGACCGTGAGACTGGAGACACGC	553	30
TGFβ1	GTAGTTGTGGACAGACATTGGGCGTGT	TTGTAAAGCACCATGACTTCCTCGGGC	433	28
IL-10	CACAGACTTCGGTAATGCGCTCTCCAG	ACCATTGTCCTGCATCTTGCTGTAGGTT	403	32
Arg1	CCATGTCACTGAGGAGCAACTTCGTGC	TGCAGTTCTCTCAGCAGGAACGACACA	523	28
